# Introductory Lectures and Addresses on Medical Subjects

**Published:** 1860-05

**Authors:** 


ART. VI.—*Introductory Lectures and Addresses on Medical Subjects, delivered chiefly before the Medical Classes of the University of Pennsylvania.* By GEORGE B. WOOD, M.D., LL.D. 8vo., pp. 460. Philadelphia: J. B. Lippincott & Co., 1859.

THE present volume will no doubt prove a source of great gratification to the many friends of Professor Wood, especially the graduates of the University of Pennsylvania, to whom they are affectionately dedicated by their distinguished author. It was well that, upon his retirement from an institution which he so long adorned with his scholastic labors, and of which he was one of the noblest pillars, he should have reproduced these memorials of his zeal and devotion to the interests of his pupils, uttered before them on various occasions either as he met them for the first time in the lecture-room, or as he greeted them for the last time on commencement day. Their perusal will awaken sad reflections in the minds of many readers, when they consider that the voice to which they were wont to listen with so much delight and profit is to be no longer heard in the halls of their Alma Mater; that he will no more stand before his pupils upon the rostrum as an oral instructor; and that his honored name will henceforth appear only upon annual announcements and catalogues of the school in connection with the title of Emeritus Professor.

But while they thus grieve for the loss of their teacher, the friends and graduates of the University of Pennsylvania have much to console them. Dr. Wood retires from his scholastic duties in the vigor of his manhood, ere the hand of mental and physical decay has made the slightest perceptible impress; at a moment when he lectured with as much force, and ability, and precision, as when he entered upon his career a quarter of a century ago. He might with great propriety have remained in the occupancy of his chair a number of years longer; but, unlike some selfish spirits with whom it was once our lot to be associated, he has chosen to retire in the full consciousness of his power, and amidst the profound regrets of every right-thinking man in the profession. His example is worthy of all imitation. There is a time when a professor, like a general or a statesman, ought to retire from public life, and that time unquestionably is when his mental and physical faculties are in their full vigor; not when he is tottering under the effects of old age, when he has to be lifted into and out of his chair, and when his usefulness as an instructor has departed from him never to return.

It is difficult to determine when a man holding an important office should be legally superannuated, or regarded as unfit for the exercise of his duties. The Legislature of New York did, perhaps, a wise thing when it passed a law declaring that her public officers should retire at sixty-five; and yet Chancellor Kent, whom this enactment was, if we mistake not, particularly designed to affect, was not only in the full possession of his intellectual and physical functions at the time of his resignation, but he delivered for many years afterwards annual courses of lectures on law, and composed his immortal commentaries, one of the great works of the age, characterized by remarkable erudition and elegance of style. Caldwell remained in his chair until he was compelled to vacate it by an act of the board of trustees of the University of Louisville; and Russell, of Edinburgh, also an octogenarian, left that celebrated school only after he had been bought off! Age and inability are, of course, relative terms. A man may be old and worthless at forty, while another may be young and in full vigor, both of mind and body, at seventy or even eighty. Chief Justice Marshall retained all his manly vigor until death freed him alike of life and of office; and Lord Brougham, an octogenarian, is still in full possession of all his mental faculties. General Cass is rapidly approaching his eightieth year, and yet he daily performs with great ability all the duties of his important and laborious office of Secretary of State at Washington. The "Iron Duke," at the same age, was never in better trim, or more wise in council, and more resolute in action. We do not believe that young men are by any means the best teachers; to give instruction in any branch of medical science, in any degree commensurate with the wants and requirements of the student, demands vastly more preparation and an amount of knowledge which few young men, under any circumstances, can command. A ripe judgment and a long experience are always most efficient aids in every well-matured course of lectures; but there is, nevertheless, a period beyond which a professor ought not, as a general rule, to continue in office. His self-respect, if nothing else, should prompt him to quit public life the moment he becomes conscious that he can no longer discharge the duties of his chair with credit to himself, fidelity to his class, and honor to the institution with which he is connected.

Dr. Wood has been an eminently successful and fortunate man. Providence has heaped upon him its choicest blessings. All the honors that a medical man can achieve he has received, and, more than all, he has well deserved them. For twenty-five years a professor in one of the most renowned medical schools in the world, at first of materia medica, and afterwards of medicine, he was for a long time physician to the Pennsylvania Hospital, and for many years he has been President of the College of Physicians, as he was formerly of the American Medical Association, and as he is now of the American Philosophical Society. Some years ago Princeton College conferred upon him the degree of

LL.D. As a writer he ranks deservedly high. His works on Materia Medica and the Practice of Medicine, and his labors as one of the authors of the United States Dispensatory, an immense storehouse of erudition, published jointly by himself and Professor Bache, have given him a world-wide reputation among medical men.

The volume before us is issued in a superior style, highly creditable to the liberality of the publishers, and the taste of Dr. Wood. It comprehends a great deal of useful matter, entitling it to a place in every medical library.

